# Global inorganic nitrogen dry deposition inferred from ground- and space-based measurements

**DOI:** 10.1038/srep19810

**Published:** 2016-01-27

**Authors:** Yanlong Jia, Guirui Yu, Yanni Gao, Nianpeng He, Qiufeng Wang, Cuicui Jiao, Yao Zuo

**Affiliations:** 1 Synthesis Research Center of Chinese Ecosystem Research Network, Key Laboratory of Ecosystem Network Observation and Modeling, Institute of Geographic Sciences and Natural Resources Research, Chinese Academy of Sciences, Beijing 100101, China; 2 University of Chinese Academy of Sciences, Beijing 100049, China; 3 State Key Laboratory of Environmental Criteria and Risk Assessment, Chinese Research Academy of Environmental Sciences, Beijing 100012, China

## Abstract

Atmospheric nitrogen (N) dry deposition is an important component in total N deposition. However, uncertainty exists in the assessment of global dry deposition. Here, we develop empirical models for estimating ground N concentrations using NO_2_ satellite measurements from the Ozone Monitoring Instrument (OMI) and ground measurements from 555 monitoring sites. Global patterns and trends in the fluxes of NO_2_, HNO_3_, NH_4_^+^, and NO_3_^−^ were assessed for 2005–2014. Moreover, we estimated global NH_3_ dry deposition directly using data from 267 monitoring sites. Our results showed that East Asia, the United States, and Europe were important regions of N deposition, and the total annual amount of global inorganic N deposition was 34.26 Tg N. The dry deposition fluxes were low in Africa and South America, but because of their large area, the total amounts in these regions were comparable to those in Europe and North America. In the past decade, the western United States and Eurasia, particularly eastern China, experienced the largest increases in dry deposition, whereas the eastern United States, Western Europe, and Japan experienced clear decreases through control of NO_x_ and NH_3_ emissions. These findings provide a scientific background for policy-makers and future research into global changes.

Rapid industrial development and agricultural production emit large quantities of NO_x_ (NO + NO_2_) and NH_3_ to the atmosphere^1^,^2^. After a series of chemical transformations and physical transport processes in the atmosphere, NH_3_ and NO_x_ are removed through dry deposition and wet scavenging and deposited on the Earth’s surface^3^,^4^. Due to the ease of observation, wet nitrogen (N) deposition has been widely and intensively researched^5^,^6^. However, growing evidence suggests that dry N deposition is also an important component in total N deposition. For example, Pan *et al.* reported that dry deposition represents approximately 60% of the annual total deposition, according to the average results of ten sites in northern China^7^. Gaseous N and particulate N (two forms of dry deposition) can cause a series of biochemical reactions after they penetrate leaves through plant stoma or directly enter the soil^8^. These reactions then affect the structure and function of an ecosystems^9^. Therefore, quantifying the spatio-temporal patterns of dry deposition is critical to comprehensively understanding the role of dry deposition in global N cycles and to providing scientific background on its ecological effects.

The methods for measuring dry deposition fluxes at the site scale include the eddy correlation method, chamber method, inferential method, and gradient method^10^. The inferential method is currently the most common method because of its easy operation, low cost, and relatively high accuracy^11^,^12^. In this method, ground air N concentrations and deposition velocities (V_d_) are key factors in the calculation of dry deposition fluxes. Currently, the primary methods for sampling ground air N concentrations include filter packs, denuders, and passive samplers, and the chemical methods for measuring air N are the ion chromatography and spectrophotometric methods. Although many different methods for sampling and chemical measurement exist, the results from different methods are highly consistent^13^,^14^,^15^. V_d_, the other key factor of dry deposition, is usually simulated using models such as a big leaf model^16^,^17^,^18^ and the Multi-Layer Model^19^. The observation of sites in different regions worldwide provides the basis to evaluate and validate N deposition at global or regional scales^2^,^18^.

The main methods for evaluating dry deposition fluxes at regional or global scales are geostatistical methods and model simulation. For example, the geostatistical method has been used to evaluate the spatial patterns of dry deposition in Europe, the United States and China^5^,^18^; Dentener *et al.*^3^ and Vet *et al.*^2^ simulated global total N deposition including dry and wet forms based on multiple atmospheric chemistry transport models. Recently, a new method was developed to study the spatial pattern of dry deposition by applying satellite observations^20^,^21^. Cheng *et al.* used Global Ozone Monitoring Experiment (GOME) and Scanning Imaging Absorption Spectrometer for Atmospheric Chartography (SCIAMACHY) observations to determine the spatial and temporal characteristics of NO_2_ dry deposition based on the empirical relationship between NO_2_ columns and rural NO_2_ *in situ* measurements in eastern China^20^. Nowlan *et al.* characterized global NO_2_ dry deposition fluxes using satellite measurements from the Ozone Monitoring Instrument (OMI) in combination with simulations from the GEOS-Chem model^21^. This new method features certain major advantages: First, satellite observations can be used to evaluate spatially and temporally continuous NO_2_ fluxes^20^,^21^. Second, these observations can provide results with a higher spatial resolution than model simulations at the global scale^21^. Third, fewer parameters are needed in this method than in the model simulations^20^. Consequently, it is worthwhile to develop a theory and methodology for evaluating the spatio-temporal patterns of global dry deposition using satellite observations.

This study compiled a worldwide dataset of atmospheric inorganic N concentrations from 555 ground monitoring sites (Fig. 1), including 7,424 site-year data, downloaded OMI NO_2_ column standard products between 2005 and 2014, and data on dry deposition velocities from 163 sites worldwide. Based on the chemical transformations between airborne reactive N, we developed methods that can, for the first time, determine the 2005—2014 global patterns and trends in dry deposition fluxes directly from ground- and space-based data.

## Results

### Spatial patterns of global dry N deposition fluxes

The magnitude and spatial patterns of global dry deposition fluxes differed significantly by region and N species (Fig. 2). In summary, eastern China, Western Europe, and the eastern United States were the three global hotspots for NO_2_, HNO_3_, NH_4_^+^, and NO_3_^−^ fluxes. According to the site results for NH_3_ fluxes (Fig. 2e; for details, see Methods), high flux values occurred in China, India, and North Africa, whereas lower flux values were present in Europe and the United States. The global averages of the NH_3_, NO_2_, HNO_3_, NH_4_^+^, and NO_3_^−^ fluxes were 1.64, 0.45, 0.27, 0.11, and 0.02 kg N ha^−1^ a^−1^, respectively. The highest values for total N fluxes, including five N species and with values of approximately 50–55 kg N ha^−1^ a^−1^, occurred in eastern China.

### Trend analysis of global N dry deposition fluxes

The average annual changes in the dry deposition fluxes (the sum of the NO_2_, HNO_3_, NH_4_^+^, and NO_3_^−^ fluxes) ranged from −1.9 to 2.0 kg N ha^−1^ a^−1^ between 2005 and 2014, with a global mean value of 0.018 kg N ha^−1^ a^−1^ (Fig. 3). These results indicated that the dry deposition fluxes increased or decreased in some regions annually and exhibited a weak positive trend worldwide. The significant increases were located in the western United States and Eurasia, particularly eastern China, and the significant decreases occurred in the eastern United States, Western Europe, and Japan. Globally, HNO_3_ was the most abundant N species in the dry deposition flux increases because of its high deposition velocity, followed by NO_2_, NH_4_^+^, and NO_3_^−^.

### Global and regional total dry N deposition

Table 1 shows the global and regional total dry deposition. Based on the results inferred from the OMI NO_2_ columns, NO_2_ was the most abundant N species in dry deposition globally, followed by HNO_3_, NH_4_^+^, and NO_3_^−^. Asia and Africa received the largest volume of deposition, based on the sum of these four N species (“Subtotal” in Table 1), followed by North America, South America, Europe, and Oceania. Asia and Africa were also the regions with the greatest deposition based on a single N species.

In the present study, the regional deposition of NH_3_ was calculated as the product of the averaged fluxes based on site measurements and a regional area. Because crop sites represented a large portion of the collected NH_3_ sites (approximately 1/3), the regional NH_3_ deposition results in this study may be overestimated. According to our results (Table 1), the global deposition of NH_3_ was 22.28 Tg N a^−1^, and Asia and Africa were the regions with the greatest deposition, followed by South America, North America, Europe, and Oceania. Summing all five N species, the global total deposition was approximately 34.26 Tg N a^−1^.

## Discussion

### Dry deposition and dry/wet deposition ratios from different studies

Studies on dry deposition at a large scale are still limited, and they have primarily focused on regions with high N deposition, i.e., the United States, Europe, and China. The results of this study and previous studies on both global- and regional-scale dry deposition are listed in Table 2. Our results are comparable to previous studies, and the large-scale dry deposition results that differ are on the same order of magnitude (Table 2). Additionally, certain differences exist among the results from different methods because each method has its own uncertainty. The uncertainty in the atmospheric chemistry transport models is primarily derived from the assessment of NO_x_ and NH_3_ emissions and the dry deposition parameterizations^2^,^3^. The accuracy of geostatistical methods depends on the number, distribution and types of monitoring sites^5^,^18^. However, globally, N deposition monitoring sites are still rare except in Europe, North America, and Asia^2^. The method used in the present study is based on data from ground and satellite measurements, and the uncertainty in this method arises from these two data sources. Although all types of methods have their own uncertainties, the development of multiple methods will make the evaluation of dry deposition more accurate and comprehensive. Compared to other methods, the method used in this study has two advantages. First, this method has a simpler structure and requires fewer parameters, which reduces computation time and decreases uncertainty associated with the multiple data sources. Second, this method can conveniently provide continuous results for trend analysis of dry deposition.

Based on previous studies of wet deposition (Table 2), we calculated dry/wet deposition ratios in the United States, Europe, and China and found the average ratios to be 0.93, 0.55, and 0.56, respectively. In the United States, Europe, and China, dry deposition contributed 48%, 35%, and 36% to total deposition, respectively. Vet *et al.* estimated that the global total deposition was 79.5 Tg N a^−1^ based on multiple models^2^. However, they did not note the specific value of dry deposition. If we assume that 40% of the total deposition is deposited via dry deposition, then the global dry deposition would be 31.8 Tg N a^−1^ according to the total deposition result from Vet *et al.* This value is close to our result of 34.26 Tg N a^−1^. The above analysis corroborated dry deposition’s important role in global N deposition. However, the majority of ecological field experiments on N enrichment to date have focused on wet deposition fluxes; therefore, the investigation of how dry deposition affects ecosystem structures and functions is an important ecological issue.

### Key hotspots of dry deposition changes

According to the results of our trend analysis between 2005 and 2014 (Fig. 3), the eastern United States, Western Europe, and Japan show a clear declining trend in dry deposition, corresponding to monitoring site reports from the United States and Europe^6^,^22^. These findings suggest that dry deposition is still high in these regions but has declined significantly in recent years. As a result of the Cross-State Air Pollution Rule, NO_x_ emissions from electrical generation are expected to have decreased by over 50% from 2005 to 2014 in the eastern United States^23^. In the 28 EU countries, NO_x_ emissions and NH_3_ emissions decreased on average 51% and 28%, respectively, from 1990 to 2012 through the control of air pollution emissions^22^. These policy examples suggest that N deposition can clearly be decreased by controlling NO_x_ and NH_3_ emissions, which is important for weakening the potential detrimental effects of N saturation on ecosystems^24^,^25^,^26^.

In sharp contrast to the above regions, eastern China not only experienced high dry deposition fluxes but also featured the greatest increase in dry deposition fluxes over the past decade (Fig. 3) and the most expected hotspots of N deposition. These results agree with the continuous measurements of wet and dry deposition at ten sites in this area^7^. According to those results, the total N deposition at the ten sites ranged from 28.5 to 100.4 kg N ha^−1^ a^−1^, with an average value of 60.6 kg N ha^−1^ a^−1^. Of this total, 40% was deposited via precipitation, and the remaining 60% was deposited by dry deposition. Large NO_x_ and NH_3_ emissions are the reason for the ongoing high N deposition in this region. Between 1980 and 2010, NO_x_ and NH_3_ emissions in China grew approximately linearly and increased from 1.4 to 6.3 Tg N a^−1^ and from 5.7 to 14.5 Tg N a^−1^ (ref.^27^), respectively, resulting in inevitably high quantities of deposited N. Although N deposition can increase ecosystem carbon sequestration to a certain extent^28^, excessive N results in negative impacts on soil, water, and biological diversity^24^,^25^,^26^. In recent years, worsening smog-related weather conditions in China have created a threat to public health, and the Chinese government enacted pollution control and management regulations and strengthened measures to control pollutant emissions. N deposition is expected to decrease with the promulgation and implementation of these regulations.

### Scientific basis for establishing remote sensing empirical models

In this study, we established remote sensing empirical models to estimate ground NO_2_, TNO_3_ (the sum of HNO_3_ and NO_3_^−^), and NH_4_^+^ concentrations using OMI satellite measurements and ground measurements. Although they are empirical models, there is a scientific basis for establishing them. The logical framework for the method of determining dry N deposition is shown in Fig. 4.

Blond *et al.* noted that NO_2_ ground measurements performed in urban areas cannot be used to validate remote sensors with relatively low spatial resolutions due to strong concentration gradients in urban areas^29^. However, NO_2_ ground concentrations at rural sites, where measurements can represent large areas, are significantly positively correlated with NO_2_ columns. Based on this positive correlation, Cheng *et al.* established a remote sensing empirical model to estimate NO_2_ dry deposition in eastern China^20^. We improved Cheng’s model by developing a global NO_2_ model and modifying the parameterization and validation methods.

When NO_2_ is released into the atmosphere, it is converted into gaseous HNO_3_ or particulate NO_3_^−^ (ref. 4). The conversion processes of NO_2_ to HNO_3_ or NO_3_^−^ and the processes of mutual conversion between HNO_3_ and NO_3_^−^ are shown in Fig. 4. Because NO_2_ is the source of HNO_3_ and NO_3_^−^, we inferred that a positive relationship should exist between the number of sources and sinks, and our study demonstrated this assumption. We tested the relationships using data from monitoring sites observing the concentrations of NO_2_, HNO_3_, and NO_3_^−^ and found that NO_2_ concentrations have a strong positive correlation with the sum of HNO_3_ and NO_3_^−^ concentrations (Supplementary Fig. S1). This is the scientific basis of the TNO_3_ model.

In this study, the NH_4_^+^ model was the result of an initial attempt, but validation subsequently indicated that this model is reliable. We surmise that this model can evaluate ground NH_4_^+^ concentrations using NO_2_ columns because NH_4_NO_3_ is the main form of NO_3_^−^ in aerosols and because NO_2_ is the source of the NO_3_^−^ in NH_4_NO_3_ (see Fig. 4). Thus, a strong linear positive correlation exists between NH_4_^+^ and NO_3_^−^ concentrations^30^. Additionally, we also attempted to establish an NH_3_ model using NO_2_ columns, but the result was not satisfactory. The main reason for this is that NH_3_ and NO_2_ come primarily from agricultural and industrial activities, respectively; thus, no restrictive relationships exist in their chemical transformation due to their different sources.

### Uncertainty analysis

Although our findings are reliable based on the site data validation (see Supplementary Fig. S2), they are still uncertain to some extent. There are four possible contributors to the uncertainty. First, some error is from the OMI NO_2_ column products, derived mainly from the calculation of air mass factors (AMF), and the uncertainty in the AMF is approximately 10–40% (ref.^31^). Second, error may come from ground monitoring data. The monitoring data collected in this study were derived from different monitoring networks or the literature, and some errors may arise from researchers using different methods of sample collection and different measuring instruments. Third, some uncertainties are attributed to the estimation of deposition velocity, and previous studies have suggested that there is great uncertainty in this estimation^11^,^18^. Here, we attempt to reduce this uncertainty by collecting deposition velocity values from the published literature instead of calculating them directly. Furthermore, the regional assessment of NH_3_ deposition in our study contains uncertainties. The regional deposition of NH_3_ was calculated from the site-based NH_3_ fluxes averaged over the region, and crop sites represented a large proportion of the collection sites (approximately 1/3). Thus, the regional results of NH_3_ deposition in this study may be overestimated. We note that NH_3_ columns were retrieved from the IASI satellite^32^,^33^, and we expect that it can be used to calculate NH_3_ ground concentrations in the future. This calculation will be helpful in evaluating spatial patterns of ground-level NH_3_ concentrations more precisely, thus improving the spatial resolution of NH_3_ dry deposition.

## Methods

### Ground-level *in situ* measurements

The atmospheric inorganic N concentration data are from three sources: results published after 2000 (see the Supplementary Information for a complete reference list), N deposition monitoring networks worldwide, and the World Data Centre. Nine monitoring networks provided available data for this study: the Co-operative Programme for Monitoring and Evaluation of the Long-Range Transmission of Air Pollutants in Europe (European Monitoring and Evaluation Programme, EMEP); the Clean Air Status and Trends Network (CASTNET); the Air Quality System (AQS) and Ammonia Monitoring Network (AMoN) in the United States; the Canadian Air and Precipitation Monitoring Network (CAPMoN) and National Air Pollution Surveillance Program (NAPS) in Canada; the Acid Deposition Monitoring Network in East Asia (EANET); the Igac Debits Africa program (IDAF); and the Department of Environment and Heritage Protection of Queensland, New South Wales, and Northern Territory in Australia. Currently, the most common methods for sampling ground air N concentrations include filter packs, denuders, and passive samplers, and the chemical methods for measuring air N are the ion chromatography and spectrophotometric methods. Although many different methods for sampling and chemical measurement exist, the results from different methods are highly consistent^13^,^14^,^15^. This agreement is the basis for our analysis of the global data from different studies. To study global dry deposition at the annual scale, the criteria for collecting data were as follows. First, the land use type of the monitoring site must be clearly described, e.g., forest, crop, grassland, wetland, etc. Second, we imposed no restrictions on the sampling and measuring methods, but the sampling frequency must have been on the day, week, or month scale, and the sampling period must have been longer than one year. Third, the atmospheric concentrations of one or several species, i.e., NO_2_, NH_3_, HNO_3_, NH_4_^+^, and NO_3_^−^, must have been measured. After we collected the data, certain processes were performed to make the data available, including data collation, data unit transformation, and abnormal value elimination.

**Figure 1:**
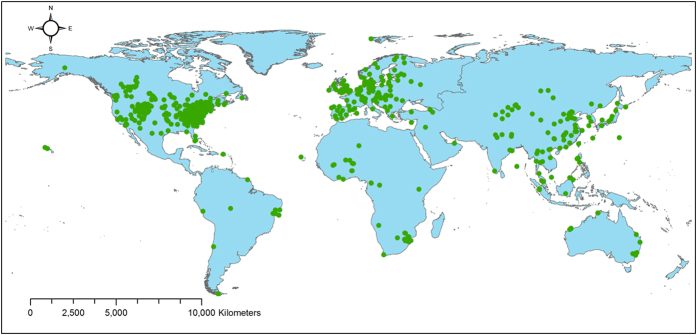
Global distribution of monitoring sites for atmospheric inorganic N concentrations. Note: At least one species of inorganic N was measured at each site. The map was generated using ArcGIS 10.0 software.

**Figure 2:**
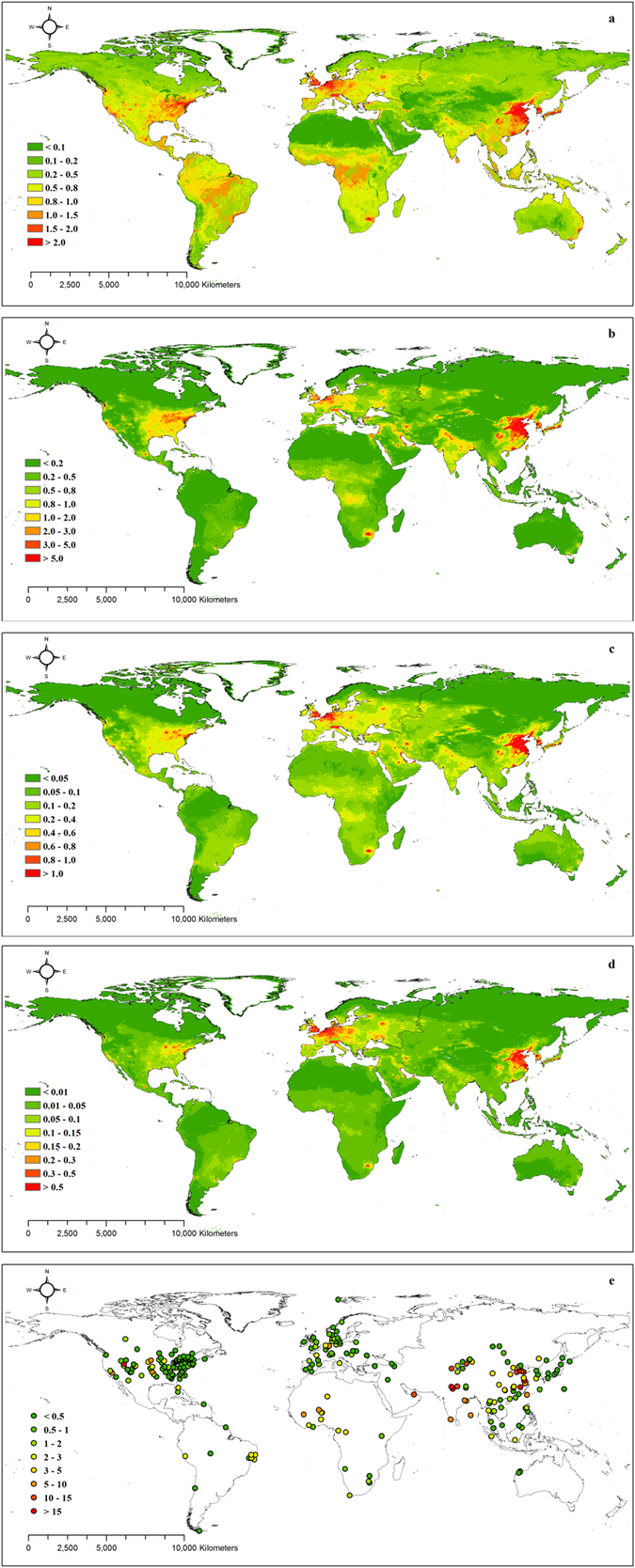
Spatial patterns of dry N deposition fluxes (kg N ha^−1^ a^−1^) for **(a)** NO_2_, (**b**) HNO_3_, (**c**) NH_4_^+^, (**d**) NO_3_^−^, and (**e**) NH_3_. The NH_3_ fluxes were derived from 267 monitoring sites (see Methods). The maps were generated using ArcGIS 10.0 software.

**Figure 3:**
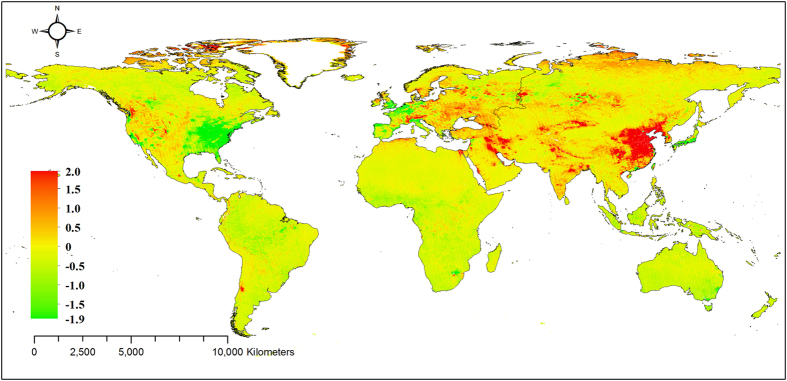
The average annual changes in dry N deposition fluxes (kg N ha^−1^ a^−1^). Note: The slope was calculated by plotting annual dry deposition fluxes against time from 2005 to 2014 in each grid, and the slope value was used to represent the annual change rate. Due to the lack of continuous data for NH_3_ fluxes, the trend analysis of dry deposition only included annual changes of NO_2_, HNO_3_, NH_4_^+^, and NO_3_^−^ fluxes. The map was generated using ArcGIS 10.0 software.

**Figure 4:**
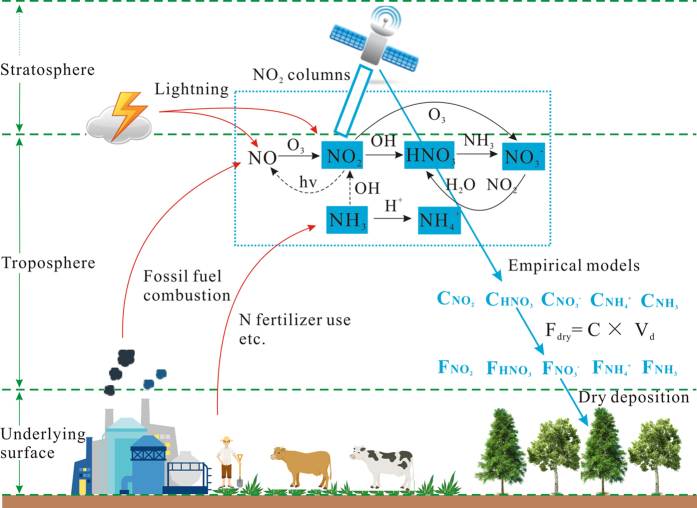
Logical framework for the method used to determine dry N deposition and atmospheric N-related processes, including N emissions and chemical transformation processes. Based on chemical transformations between inorganic N species, the OMI NO_2_ columns were used to estimate the ground concentrations of NO_2_, TNO_3_ (HNO_3_ + NO_3_^−^), and NH_4_^+^ by establishing remote sensing empirical models, and then dry deposition fluxes were calculated using the inferred method. Note: The red arrows represent N emissions from natural and anthropogenic sources; the black arrows represent the chemical transformation processes between atmospheric inorganic N species, which are discussed in the literature^4^,^10^; the solid and dotted black arrows are the primary and secondary processes, respectively; the blue arrows represent the logical framework for the evaluated method in this study; and the symbols “F”, “C”, and “V_d_” represent dry deposition flux, ground concentration, and deposition velocity of inorganic N species, respectively.

Our datasets included the following: the name of the monitoring site, location of the monitoring site, monitoring period, monitoring method, land use type, NO_2_-N concentration, NH_3_-N concentration, HNO_3_-N concentration, NH_4_^+^-N concentration, NO_3_^−^-N concentration, and the literature source. After rigorous data screening and quality control, we obtained a total of 555 sites and 7,424 site-year data for atmospheric inorganic N concentrations. There are 265 sites in North America, 124 in Europe, 98 in Asia, 32 in Africa, 23 in Oceania, and 13 in South America, and 1,588, 1,015, 1,692, 1,437, and 1,692 site-year data for NO_2_, NH_3_, HNO_3_, NH_4_^+^, and NO_3_^−^ concentrations, respectively. The monitoring sites were distributed worldwide (Fig. 1) and among the major terrestrial ecosystems, including forest, grassland, crops, shrub, desert, wetland, and tundra.

### NO_2_ columns from the OMI satellite instrument

The Ozone Monitoring Instrument (OMI) aboard the Aura satellite was launched on 15 July 2004. Aura flies in a sun-synchronous polar orbit at an altitude of approximately 705 km with a local equatorial overpass time between 13:40 and 13:50 local time. The OMI has three spectral channels with a spectral range between 270 and 500 nm and is used to measure trace gases, including O_3_, NO_2_, SO_2_, HCHO, BrO, and OCIO. It has a spatial resolution of 13 km×24 km and provides nearly global coverage every day. The details of the OMI can be obtained in Levelt *et al.*^34^.

NO_2_ vertical tropospheric columns are derived from the DOMINO v2.0 OMI NO_2_ product provided by the Tropospheric Emission Monitoring Internet Service (TEMIS, www.temis.nl). The details of this product can be found in Boersma *et al.*^31^. The unit of this product is 10^15^ molec./cm^2^ with a spatial resolution of 0.125° × 0.125°. In this study, we downloaded the global monthly product of NO_2_ columns between January 2005 and December 2014 in the format of an ESRI grid. Then, the annual NO_2_ column mean was calculated by averaging the monthly NO_2_ columns.

### Dry deposition fluxes

In the inferential method^12^, the dry deposition flux (F_dry_) is typically estimated by multiplying the atmospheric N ground concentration (C), including gaseous N and particulate N, by the deposition velocity (V_d_). The F_dry_ can be expressed by the following equation:


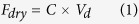


Unlike other N species, NH_3_ presents obvious bi-directional fluxes, i.e., NH_3_ can be deposited from the atmosphere onto land, but it can also be emitted from the land into the atmosphere^35^. Thus, a gaseous NH_3_ “canopy compensation point” likely exists, and deposition occurs only when the measured NH_3_ concentration is higher than the compensation point^36^,^37^. Accordingly, unlike the other four N species, the equation of F_dry_ for NH_3_ is as follows:


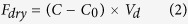


where C_0_ is the canopy compensation point of NH_3_. The values of C_0_ for various ecosystems are obtained from previous studies^38^,^39^.

According to equations (1) and (2), the calculation of F_dry_ for atmospheric inorganic N requires information on C and V_d_.

### Ground concentrations (C)

Based on global ground monitoring concentrations of atmospheric inorganic N and OMI NO_2_ columns, we developed remote sensing empirical models at an annual scale to determine the global ground NO_2_, TNO_3_, and NH_4_^+^ concentrations. Because we used the same modelling approach for these three N species, we describe the NO_2_ model as an example here. The specific approach was as follows. First, NO_2_ columns were extracted according to the locations of the monitoring sites using ArcGIS 10.0 software. The NO_2_ ground concentration at each monitoring site and the corresponding NO_2_ column were treated as a pair of data. Second, the linear model (y = a + bx) was selected as the regression model, where x was the NO_2_ column and y was the corresponding *in situ* NO_2_ ground concentration. Third, 2/3 of the pairs of data were selected to establish the model, and the other 1/3 of the data was used for model validation. Statistics of fit and validation were also calculated. Fourth, the previous step was repeated 500 times through a random and non-repeated sampling method to decrease the random error due to certain fitted data, and the averaged statistics were used to evaluate the fit and validation of the model.

In this study, the validation statistics included the coefficient of determination (R^2^), root mean square error (RMSE), and modelling efficiency (EF). The calculation and meaning of the statistics can be seen in the Supplementary Information. The final equations for estimating ground NO_2_, TNO_3_, and NH_4_^+^ concentrations are shown below (equations (3)-(5), 4, 5), and the averaged statistics of model parameterization and validation are shown in Supplementary Table S1.


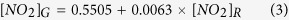



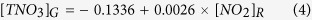



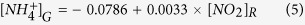


where [NO_2_]_R_ is the OMI NO_2_ column data (10^15^ molec./cm^2^) and [NO_2_]_G_, [TNO_3_]_G_, and [NH_4_^+^]_G_ are the ground measurements (μg N m^−3^) for NO_2_, TNO_3_, and NH_4_^+^, respectively. The slope and intercept values are the averaged results of 500 circulations.

The results in Supplementary Table S1 show that our models exhibited good performance in terms of model fit and validation. For example, the values of R^2^ and EF were all approximately 0.70. Based on equations (3)-(5), 4, 5 and the NO_2_ columns, we estimated the global patterns of ground NO_2_, TNO_3_, and NH_4_^+^ concentrations (Supplementary Fig. S3a-c). Because no significant correlation existed between NH_3_ ground concentrations and the NO_2_ columns, we could not establish an empirical model to estimate NH_3_ ground concentrations globally. Instead, we collected 267 NH_3_ monitoring sites from the literature and monitoring networks to assess the global pattern of ground NH_3_ concentrations. The distribution and concentration values of the NH_3_ monitoring sites are shown in Supplementary Fig. S3d.

### Deposition velocities (V_d_)

The deposition velocities of atmospheric inorganic N are from two sources: results published after 2000 and simulated results from the CASTNET network. The primary method for estimating deposition velocities in the literature is the big leaf model, and the method used in the CASTNET network is the Multi-Layer Model. A total of 163 sites containing deposition velocities were collected in this study (Supplementary Table S2). The main land uses of these sites included forest, grassland, crop, shrub, wetland, desert, and water.

Previous studies have suggested that land use was the dominant factor for dry deposition velocities^16^,^17^. The results of dry deposition velocities for different forms of N in various land uses are listed in Table 3. The results from different researchers indicated that dry deposition velocities obviously differ between different land uses. Accordingly, the average deposition velocities for the five N species in various land uses were calculated. Then, the deposition velocities of the five N species were mapped to the global land cover map according to land use types (Supplementary Fig. S4). In this present study, we used a global land cover map published by the European Space Agency (Globcover 2009)^40^ and resampled it to 0.125°×0.125°.

### Calculation and validation of dry deposition fluxes (F_dry_)

Based on the estimated global ground concentrations and the corresponding V_d_ in the above sections, we estimated the global spatial patterns of NO_2_, HNO_3_, NH_4_^+^, and NO_3_^−^ fluxes using equation (1). Because of the large difference between HNO_3_ V_d_ and NO_3_^−^ V_d_ (Supplementary Fig. S4), it was necessary to separate TNO_3_ concentrations into HNO_3_ and NO_3_^−^ concentrations to calculate their fluxes. Due to an insufficient number of monitoring sites, we separated TNO_3_ concentrations at the continental scale using the following specific method. We calculated the average HNO_3_/NO_3_^−^ ratio for each continent using monitoring sites with simultaneous observations of ground HNO_3_ and NO_3_^−^ concentrations. Using the ratios and the ground TNO_3_ concentrations, the global ground HNO_3_ and NO_3_^−^ concentrations were calculated. The average HNO_3_/NO_3_^−^ ratios were 0.60, 1.72, 1.67, 1.84, and 0.66 for Europe, Asia, North America, Africa, and South America, respectively. The ratio for Oceania was assumed to be 1.00 due to a lack of monitoring sites. Additionally, we calculated NH_3_ fluxes using equation (2) based on concentration measurements from 267 sites and their V_d_ values. Then, arithmetic averages were used to represent the magnitude of regional and global NH_3_ fluxes.

To verify the dry N deposition fluxes estimated in this study, we collected the site reported fluxes in the references or observing networks and compared the reported fluxes and corresponding simulated fluxes in this study (Supplementary Fig. S2). With the exception of NO_3_^−^ fluxes, the model fluxes of NO_2_, HNO_3_, and NH_4_^+^ showed good correlation with their reported fluxes (r ≈ 0.60). The averaged model fluxes of the four N species were close to their reported fluxes. Statistically, 71%, 70%, 78% and 62% of the model fluxes agreed within ±50% of the reported fluxes for NO_2_, HNO_3_, NH_4_^+^, and NO_3_^−^, respectively. Alternatively, we also noted that certain sites plotted far from the 1:1 line in the scatter plots (Supplementary Fig. S2), indicating that the model results were underestimated or overestimated to some extent at certain sites. Nonetheless, these findings demonstrated that the results of our model agree well with the majority of the reported results.

## Additional Information

**How to cite this article**: Jia, Y. *et al.* Global inorganic nitrogen dry deposition inferred from ground- and space-based measurements. *Sci. Rep.* **6**, 19810; doi: 10.1038/srep19810 (2016).

## Supplementary Material

Supplementary Information

## Figures and Tables

**Table 1:** Global and regional dry deposition (Tg N a^−1^).

	NO_2_	HNO_3_	NH_4_^+^	NO_3_^−^	Subtotal	NH_3_	Total
Asia	1.70	1.48	0.52	0.09	3.80	10.47	14.27
North America	0.90	0.95	0.26	0.06	2.18	0.86	3.05
Europe	0.60	0.50	0.22	0.09	1.40	0.91	2.32
Africa	1.32	0.90	0.34	0.06	2.62	7.98	10.60
South America	1.13	0.22	0.14	0.03	1.52	1.79	3.31
Oceania	0.32	0.07	0.06	0.01	0.46	0.26	0.72
Global land	5.97	4.12	1.55	0.34	11.99	22.28	34.26

Note: Dry deposition per region was calculated by multiplying the average fluxes by the regional area. “Subtotal” represents the sum of NO_2_, HNO_3_, NH_4_^+^, and NO_3_^−^ dry deposition per region. “Total” represents the summed total of the dry deposition of all five N species per region.

**Table 2:** Comparison of global and regional N deposition from different studies (Tg N a^−1^).

Reference	Method	Study region	Study period	Dry deposition					Wet deposition
				NO_2_	HNO_3_	NH_4_^+^	NO_3_^−^	NH_3_	NH_4_^+^+NO_3_^−^
Zhang *et al.* 2012^42^	GEOS-Chem model	United States	2006–2008	0.64	1.6	0.2	0.068	0.83	2.6
Holland *et al.* 2005^18^	Geostatistical method	United States	1978–1994		1.2^a^	0.18–0.98			2.36
		Europe	1978–1994	1.24	0.55–2.27^b^	0.33–1.34			6.3
Nowlan *et al.* 2014^21^	Combining remote sensing and GEOS-Chem model	United States	2005–2007	0.18					
		Europe	2005–2007	0.2					
		China	2005–2007	0.18					
		Global land	2005–2007	1.5					
Lü *et al.* 2007^5^	Geostatistical method	China	2003	2.9					9.45
This study	Remote sensing empirical model	United States	2005–2014	0.62	0.65	0.18	0.05	0.59	
		Europe	2005–2014	0.60	0.50	0.22	0.09	0.91	
		China	2005–2014	0.64	1.10	0.27	0.07	5.39	13.32^c^
		Global land	2005–2014	5.97	4.12	1.55	0.34	22.28	
Vet *et al.* 2014^2^	Atmospheric chemistry transport models	Global land	2001	79.50^d^					

Note:

^a^Represents the sum of HNO_3_ and NO_3_^−^ dry deposition.

^b^Represents the sum of HNO_3_ and NO_3_^−^ dry deposition.

^c^Data were derived from Jia *et al.*^41^.

^d^Represents the total N deposition, including dry and wet deposition, on global land.

**Table 3:** Comparison of dry deposition velocities in different land uses (cm s^−1^).

References	Land use	NO_2_	NH_3_	HNO_3_	NH_4_^+^	NO_3_^−^
Flechard *et al.* 2011^11^	Forests	0.15	1.64	3.28	0.80	1.12
	Semi-natural	0.10	0.64	0.95	0.10	0.13
	Grasslands	0.12	0.52	1.13	0.10	0.14
	Croplands	0.10	0.38	0.85	0.11	0.13
Zhang *et al.* 2004^43^	Crop	0.10	0.18	0.76	0.25	0.25
	Grassland	0.11	0.23	1.68	0.25	0.25
	Larch forest	0.11	0.20	2.43	0.27	0.27
	Coniferous forest	0.09	0.20	2.66	0.30	0.30
	Water	0.01	0.55	0.84	0.27	0.27
	Desert	0.03	0.04	1.44	0.28	0.28
	Tundra	0.07	0.20	1.57	0.20	0.20
	Tropical forest	0.10	0.23	2.33	0.32	0.32
	Prairie	0.13	0.23	1.16	0.28	0.28
Zhang *et al.* 2009^44^	Short grass	0.11	0.46	1.43	0.18	0.15
	Evergreen needleleaf trees	0.28	0.58	1.82	0.13	0.18
	Mixed forest	0.13	0.34	1.02	0.09	0.12
	Transitional forest	0.22	0.42	1.26	0.11	0.14
	Deciduous broadleaf trees	0.13	0.30	0.86	0.10	0.12
	Crops	0.07	0.32	1.02	0.15	0.14
Adon *et al.* 2013^12^	Open grassland with sparse shrub	0.15	0.22	0.69	—	—
	Deciduous shrubland with sparse trees	0.20	0.32	1.00	—	—
	Deciduous open woodland	0.20	0.35	0.98	—	—
	Mosaic forest/savanna	0.28	0.51	1.19	—	—
	Closed evergreen lowland forest	0.33	0.84	2.21	—	—
Su *et al.* 2009^45^	Urban	0.03	0.05	—	—	—
	Crop	0.07	0.12	—	—	—
	Range	0.06	0.07	—	—	—
	Larch forest	0.04	0.04	—	—	—
	Mixed forests	0.03	0.06	—	—	—
	Desert	0.02	0.02	—	—	—
	Wetland	0.02	0.33	—	—	—
	Terraces	0.07	0.10	—	—	—
	Shrubs	0.06	0.05	—	—	—
Schrader *et al.* 2014^46^	Mixed forest	—	1.50	—	—	—
	Deciduous forest	—	1.10	—	—	—
	Semi-natural	—	0.90	—	—	—
	Urban	—	0.70	—	—	—
	Water	—	0.70	—	—	—
	Agricultural	—	1.00	—	—	—
