# Supplementary material for: Global inorganic nitrogen dry deposition inferred from ground- and space-based measurements
Source: Sci Rep. 2016 Jan 27;6:19810. doi: 10.1038/srep19810 (PMC4728405; doi:10.1038/srep19810)
Supplement: Supplementary Information [file srep19810-s1.pdf]

Supplementary Information

Global inorganic nitrogen dry deposition inferred from ground- and space-based measurements

Yanlong Jia[1,2], Guirui Yu[1*], Yanni Gao[3], Nianpeng He[1], Qiufeng Wang[1], Cuicui Jiao[1,2] & Yao Zuo[1,2]

[1] Synthesis Research Center of Chinese Ecosystem Research Network, Key Laboratory of Ecosystem Network Observation and Modeling, Institute of Geographic Sciences and Natural Resources Research, Chinese Academy of Sciences, Beijing 100101, China.

[2] University of Chinese Academy of Sciences, Beijing 100049, China.

[3] State Key Laboratory of Environmental Criteria and Risk Assessment, Chinese Research Academy of Environmental Sciences, Beijing 100012, China.

*Correspondence and requests for materials should be addressed to G.Y. (yugr@igsnrr.ac.cn)

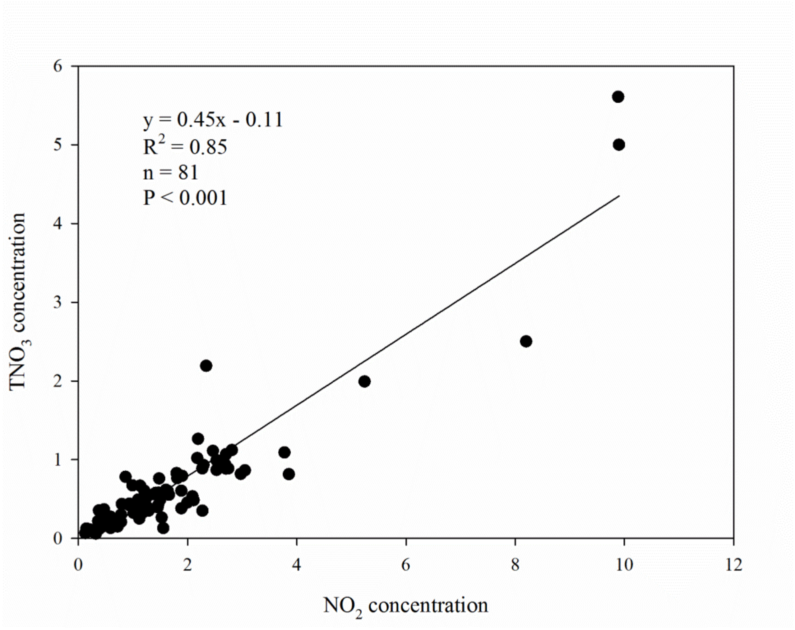

Supplementary Figure S1. Correlation between ground NO_2 concentrations and TNO_3 concentrations (μ g N m^{-3}).

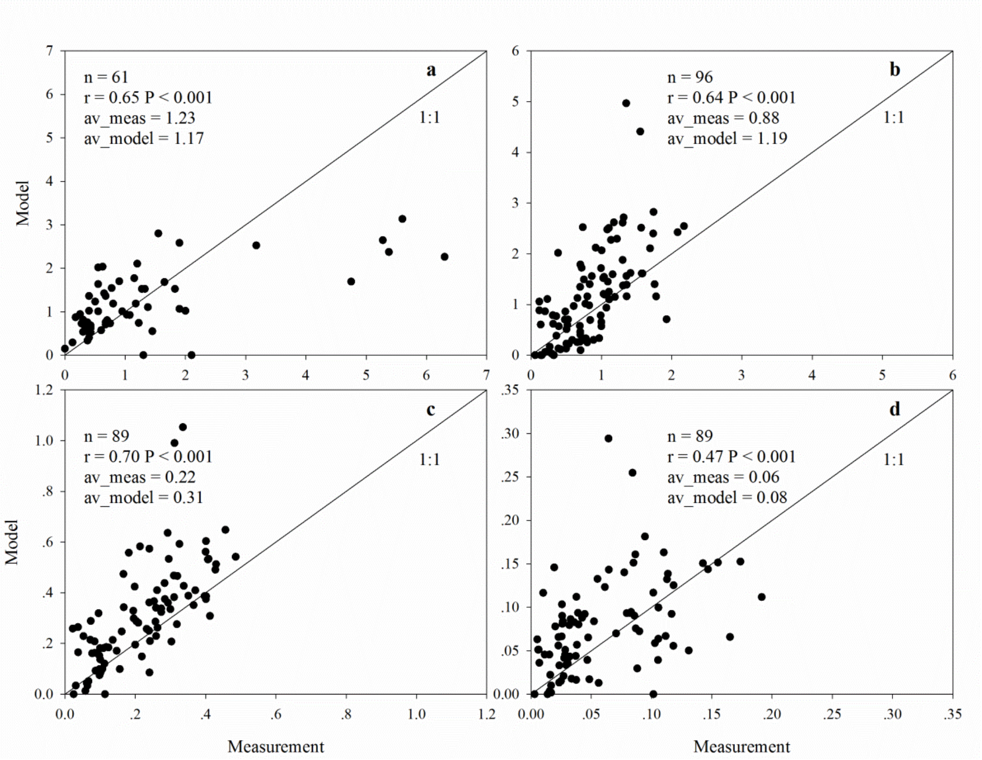

Supplementary Figure S2. Scatter plots for model dry deposition fluxes versus reported dry deposition fluxes for (a) NO_2, (b) HNO_3, (c) NH_4^+, and (d) NO_3^-.

Supplementary Tabel S1. Statistics of the empirical model parameterization and validation for atmospheric inorganic N concentrations

Models	Parameterization			Validation		
	R^2	RMSE	EF	R^2	RMSE	EF
NO_2 Model	0.69	1.35	0.69	0.68	1.37	0.65
TNO_3 Model	0.81	0.28	0.81	0.79	0.31	0.70
NH_4^+ Model	0.71	0.68	0.71	0.71	0.71	0.65

Note: there are 279, 221, and 237 pairs of data for NO_2, TNO_3 (HNO_3 + NO_3^-), and NH_4^+, respectively; 2/3 of pairs of data are selected for establishing model, and the other 1/3 of data are used for the model validation; the statistics are averaged results of 500 circulations.

The specific calculations of the statistics for model parameterization and validation are as follows.

$$R^2 = \left(\frac{\sum_{i=1}^{n}(x_i - \bar{x})(y_i - \bar{y})}{\sqrt{\sum_{i=1}^{n}(x_i - \bar{x})^2 \cdot \sum_{i=1}^{n}(y_i - \bar{y})^2}} \right) \quad (1)$$

$$RMSE = \sqrt{\frac{\sum_{i=1}^{n}(x_i - y_i)^2}{n}} \quad (2)$$

$$EF = 1 - \frac{\sum_{i=1}^{n}(x_i - y_i)^2}{\sum_{i=1}^{n}(x_i - \bar{x})^2} \quad (3)$$

(1) The coefficient of determination, R^2, represents the fraction of the variation in the observations that can be explained by the model.

(2) The root mean square error, *RMSE*, is used to measure the biases between the simulations and the observations.

(3) Modeling efficiency, *EF*, is sensitive to the systematic deviation and can represent the consistency between the observations and the simulations with range from $-\infty$ to 1. The value of EF closer to 1 indicates a more perfect match between the simulations and the observations.

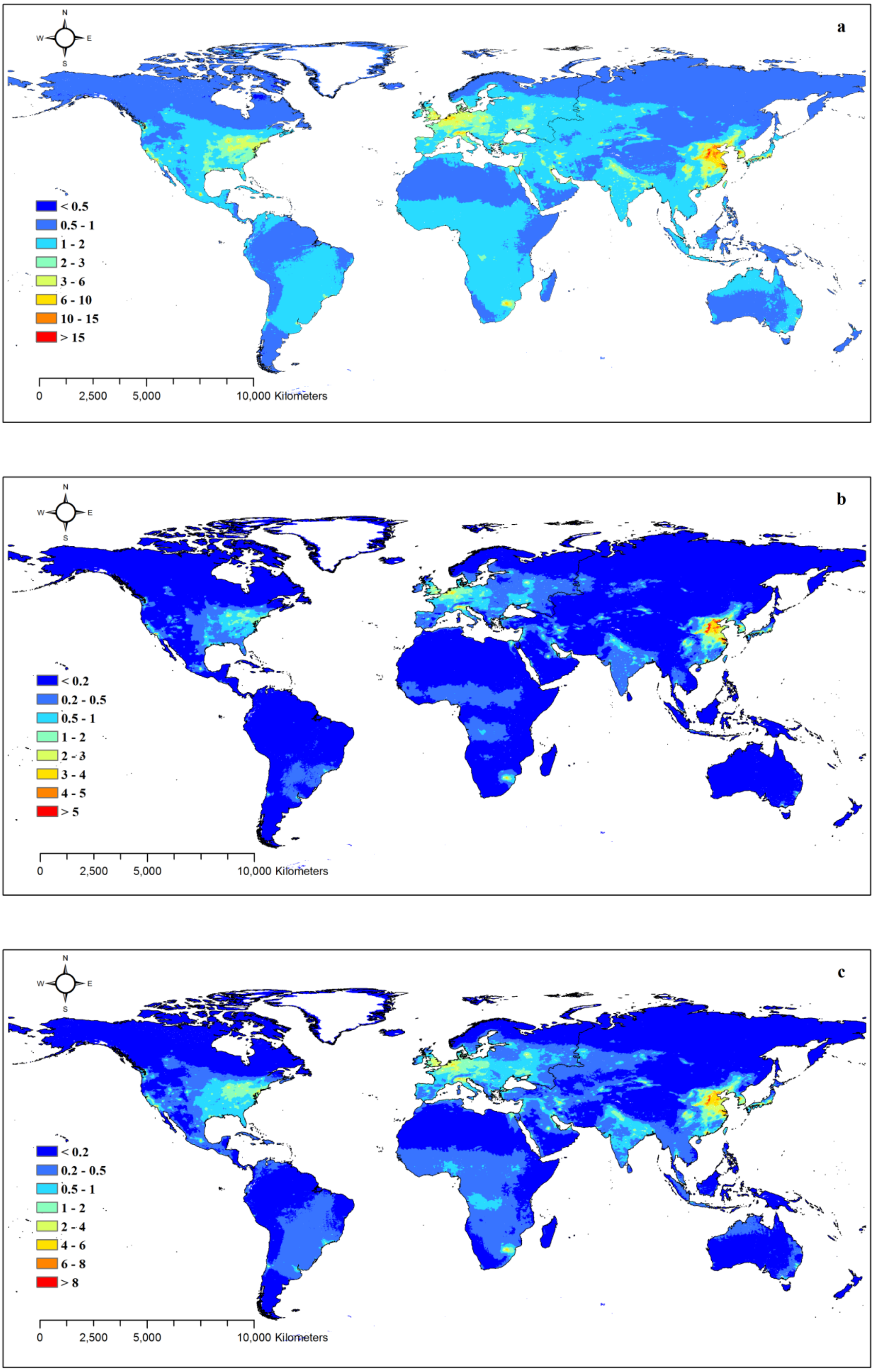
a
N
W
E
S
< 0.5
0.5 - 1
1 - 2
2 - 3
3 - 6
6 - 10
10 - 15
> 15
0
2,500
5,000
10,000 Kilometers
b
N
W
E
S
< 0.2
0.2 - 0.5
0.5 - 1
1 - 2
2 - 3
3 - 4
4 - 5
> 5
0
2,500
5,000
10,000 Kilometers
c
N
W
E
S
< 0.2
0.2 - 0.5
0.5 - 1
1 - 2
2 - 4
4 - 6
6 - 8
> 8
0
2,500
5,000
10,000 Kilometers

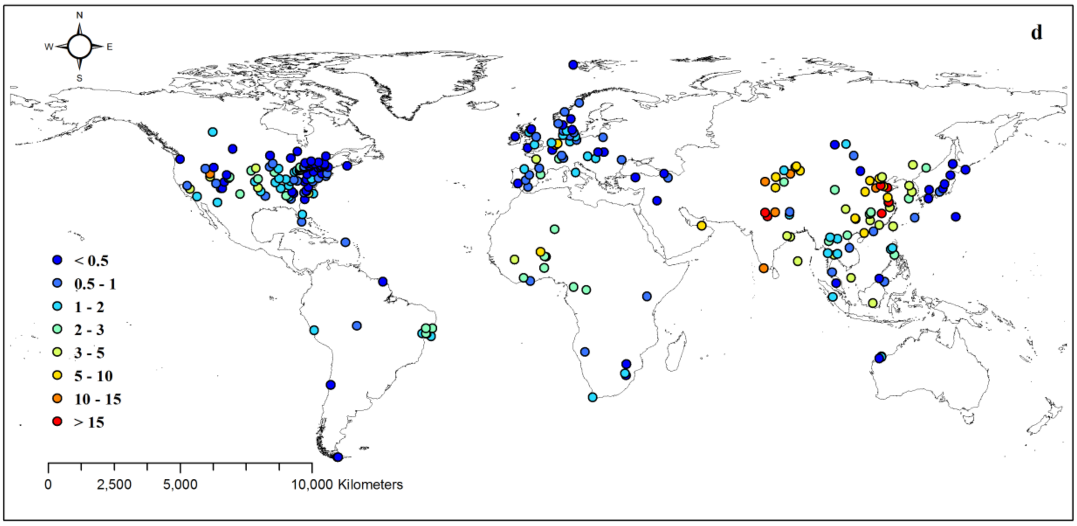

Supplementary Figure S3. Global patterns of ground atmospheric N concentrations (μg N m^{-3}) for (a) NO_2, (b) TNO_3, (c) NH_4^+, and (d) NH_3. The results of NO_2, TNO_3, and NH_4^+ derived from OMI NO_2 columns, and the results of NH_3 were directly from site observations. The maps were generated using ArcGIS 10.0 software.

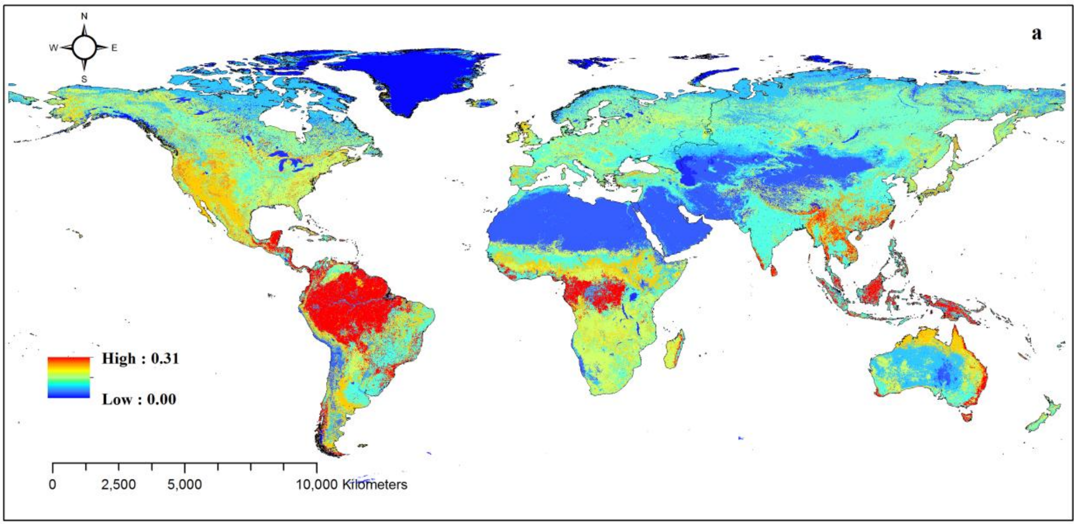
a
N
W
E
S
High : 0.31
Low : 0.00
0
2,500
5,000
10,000 Kilometers

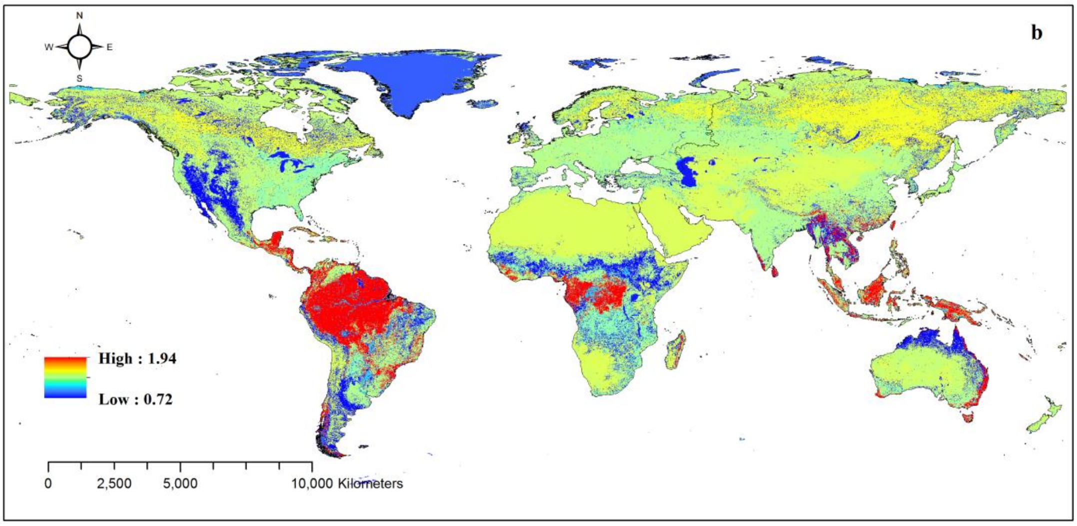
b
N
W
E
S
High : 1.94
Low : 0.72
0
2,500
5,000
10,000 Kilometers

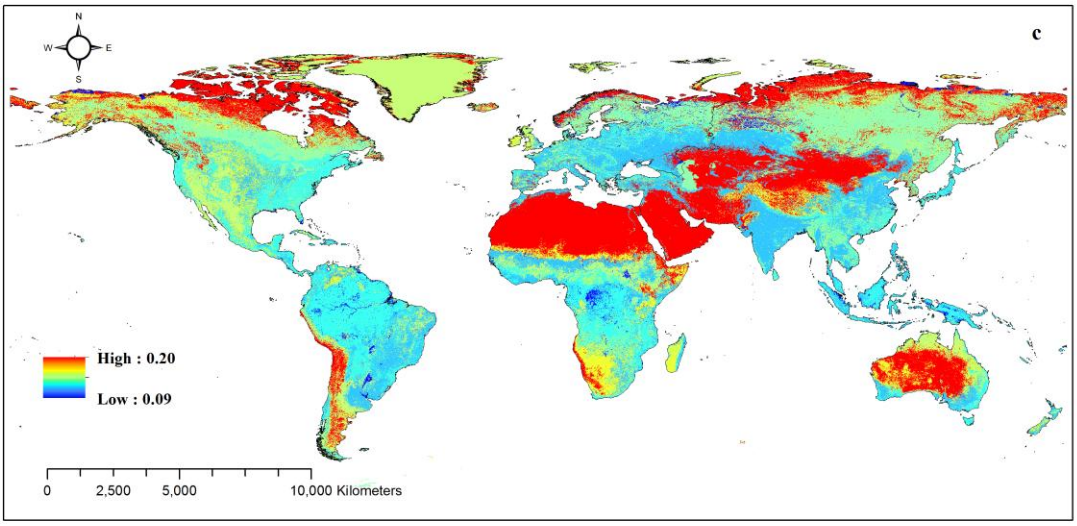
c
N
W
E
S
High : 0.20
Low : 0.09
0
2,500
5,000
10,000 Kilometers

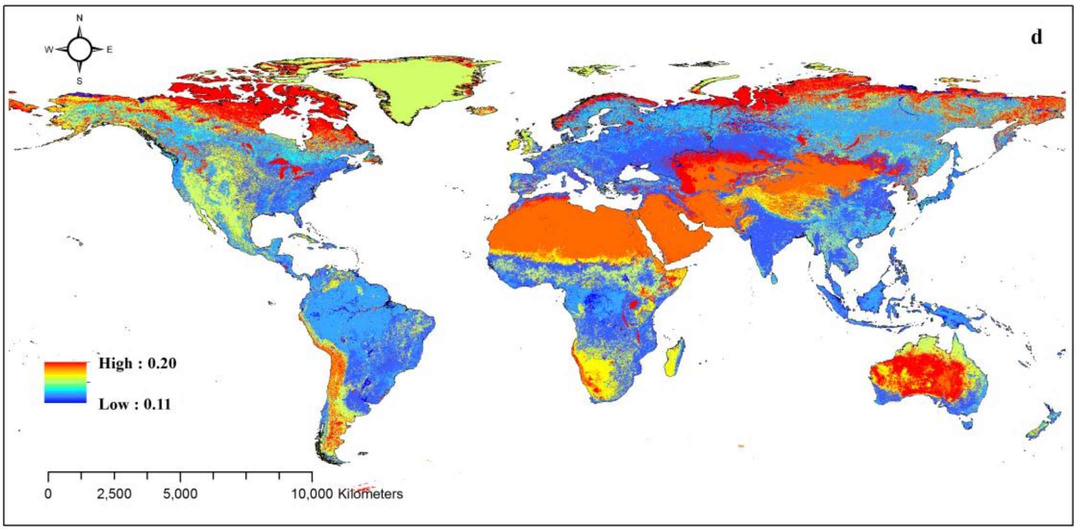

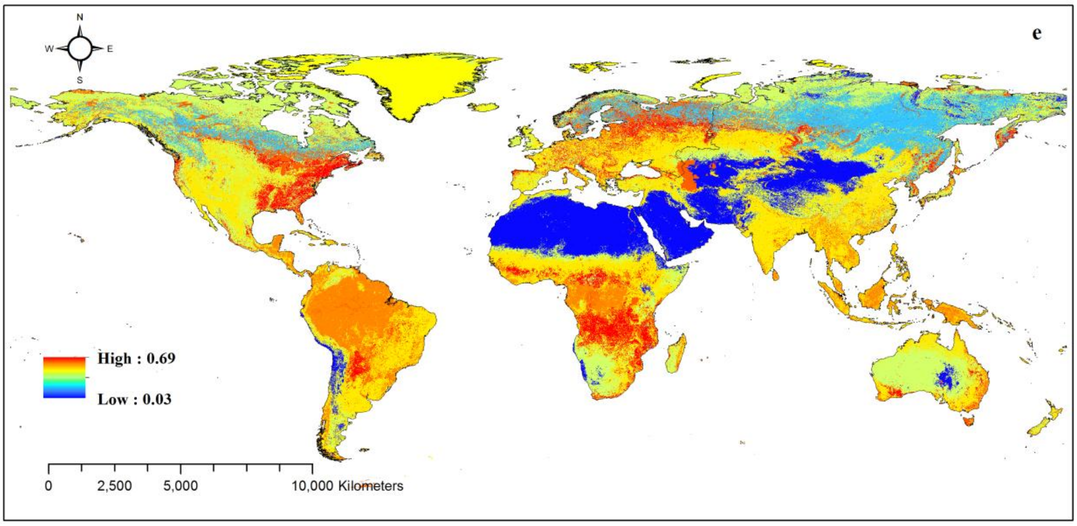

Supplementary Figure S4. Global patterns of dry deposition velocities (cm s^{-1}) for (a) NO_2, (b) HNO_3, (c) NH_4^+, (d) NO_3^-, and (e) NH_3. The maps were generated using ArcGIS 10.0 software.

Supplementary Tabel S2. Dry deposition velocities for different land uses collected from literatures

NO.	Land use	NO_2 ($cm\ s^{-1}$)	NH_3 ($cm\ s^{-1}$)	HNO_3 ($cm\ s^{-1}$)	NH_4^+ ($cm\ s^{-1}$)	NO_3^- ($cm\ s^{-1}$)	References
1	Crop	0.02-0.1	0.4-1.0	-	-	-	Pan et al., 2012
2	Crop	0.03-0.16	0.6-1.2	-	-	-	Pan et al., 2012
3	Forest	0.02-0.09	0.7-1.5	-	-	-	Pan et al., 2012
4	Arable	0.1625	-	2.06	0.1	0.26	Marner et al., 2004
5	Grassland	0.1625	-	1.39	0.06	0.15	Marner et al., 2004
6	Water	0	-	0.64	0.11	0.23	Marner et al., 2004
7	Dry and wet savannah	0.2-0.35	0.23-0.42	0.63-0.73	-	-	Delon et al., 2012
8	All land uses	0.36	0.65	2.7	0.15	0.15	Zhang et al., 2012
9	Crop	0.12	0.25	-	0.16	0.16	Cui et al., 2010
10	Short grass	0.22	0.43	1.35	0.15	0.155	Zhang et al., 2009
11	Short grass	0.11	0.46	1.43	0.18	0.15	Zhang et al., 2009
12	Evergreen needleleaf trees	0.28	0.575	1.82	0.13	0.18	Zhang et al., 2009
13	Mixed forest	0.125	0.34	1.02	0.085	0.115	Zhang et al., 2009
14	Transitional forest	0.215	0.415	1.255	0.105	0.135	Zhang et al., 2009
15	Deciduous broadleaf trees	0.13	0.3	0.855	0.1	0.12	Zhang et al., 2009
16	Evergreen needleleaf trees	0.165	0.36	0.99	0.095	0.13	Zhang et al., 2009
17	Crops	0.07	0.32	1.02	0.15	0.14	Zhang et al., 2009
18	Coastal region	-	-	-	0.028	0.095	Qi et al., 2013
19	Tea field	0.1	0.64	0.9	0.12	0.13	Shen et al., 2013
20	Paddy field	0.12	0.5	0.85	0.15	0.16	Shen et al., 2013
21	Crop	0.13	0.23	-	-	-	Yang et al., 2010
22	Residential areas	0.03	0.07	-	-	-	Yang et al., 2010

23	Open grassland with sparse shrub	0.15	0.22	0.69	-	-	Adon et al., 2013
24	Open grassland with sparse shrub	0.15	0.23	0.72	-	-	Adon et al., 2013
25	Deciduous shrubland with sparse trees	0.20	0.32	1.00	-	-	Adon et al., 2013
26	Deciduous open woodland	0.20	0.35	0.98	-	-	Adon et al., 2013
27	Mosaic forest/savanna	0.28	0.51	1.19	-	-	Adon et al., 2013
28	Closed evergreen lowland forest	0.33	0.84	2.21	-	-	Adon et al., 2013
29	Closed evergreen lowland forest	0.31	0.78	2.02	-	-	Adon et al., 2013
30	Dry savanna ecosystems	0.1-0.33	0.11-0.39	0.34-0.61	-	-	Delon et al., 2010
31	Grass	-	0.2-0.6	0.3-3.2	0.1-0.2	0.1-0.2	Endo et al., 2011
32	Semi-natural	0.10	0.64	0.95	0.10	0.13	Flechard et al., 2011
33	Grasslands	0.12	0.52	1.13	0.10	0.14	Flechard et al., 2011
34	Croplands	0.10	0.38	0.85	0.11	0.13	Flechard et al., 2011
35	Mixed forest	-	1.50	-	-	-	Schrader et al., 2014
36	Deciduous forest	-	1.10	-	-	-	Schrader et al., 2014
37	Semi-natural	-	0.90	-	-	-	Schrader et al., 2014
38	Urban	-	0.70	-	-	-	Schrader et al., 2014
39	Water	-	0.70	-	-	-	Schrader et al., 2014
40	Agricultural	-	1.00	-	-	-	Schrader et al., 2014
41	Grassland	0.25	1.1	-	-	-	Staelens et al., 2012
42	Heathland	0.25	1.50	-	-	-	Staelens et al., 2012
43	Deciduous forest	0.25	1.90	-	-	-	Staelens et al., 2012
44	Forest	0.115	0.25	-	0.2	0.2	Fan et al., 2009a
45	Farm	-	0.55	-	-	-	Bajwa et al., 2008
46	Farm	-	1.71	-	-	-	Bajwa et al., 2008
47	Grass	-	0.34	0.958	-	-	Hole et al., 2008
48	Tundra	-	0.60	1.074	-	-	Hole et al., 2008

49	Agro-ecosystem	0.12	0.26	0.81	0.16	0.16	Zhou et al., 2010
50	Agricultural site	0.13	0.23	-	-	-	Ti, 2011
51	Residential areas	0.03	0.07	-	-	-	Ti, 2011
52	Agricultural site	0.12	0.25	0.88	0.17	0.17	Cui et al., 2009
53	Crop	0.1	0.18	0.76	0.25	0.25	Zhang et al., 2004
54	Grassland	0.11	0.23	1.68	0.25	0.25	Zhang et al., 2004
55	Larch forest	0.11	0.20	2.43	0.27	0.27	Zhang et al., 2004
56	Coniferous forest	0.09	0.20	2.66	0.3	0.3	Zhang et al., 2004
57	Water	0.01	0.55	0.84	0.27	0.27	Zhang et al., 2004
58	Desert	0.03	0.04	1.44	0.28	0.28	Zhang et al., 2004
59	Tundra	0.07	0.20	1.57	0.2	0.2	Zhang et al., 2004
60	Tropical forest	0.1	0.23	2.33	0.32	0.32	Zhang et al., 2004
61	Prairie	0.13	0.23	1.16	0.28	0.28	Zhang et al., 2004
62	Urban	0.0321	0.0473	-	-	-	Su et al., 2009
63	Crop	0.0728	0.1227	-	-	-	Su et al., 2009
64	Range	0.0618	0.0729	-	-	-	Su et al., 2009
65	Larch forest	0.0395	0.0416	-	-	-	Su et al., 2009
66	Mixed forests	0.0345	0.0592	-	-	-	Su et al., 2009
67	Desert	0.0249	0.0200	-	-	-	Su et al., 2009
68	Wetland	0.0233	0.3273	-	-	-	Su et al., 2009
69	Terraces	0.0654	0.0982	-	-	-	Su et al., 2009
70	Shrubs	0.0572	0.0549	-	-	-	Su et al., 2009
71	Evergreen forest	0.115	0.2740	0.985	0.216	0.216	Fan et al., 2009b
72	Evergreen forest	0.114	0.2330	-	0.158	0.158	Fan et al., 2013
73	Grassland	0.12	0.2600	1.07	-	0.16	Deng et al., 2009
74	Urban/Agric	-	-	1.33	0.13	0.12	CASTNET

75	Forest	-	-	1.10	0.09	0.09	CASTNET
76	Prairie	-	-	1.56	0.13	0.14	CASTNET
77	Agric	-	-	1.06	0.08	0.07	CASTNET
78	Forest	-	-	1.27	0.10	0.09	CASTNET
79	Agric	-	-	1.45	0.13	0.12	CASTNET
80	Agric	-	-	1.49	0.12	0.10	CASTNET
81	Forest	-	-	1.67	0.17	0.17	CASTNET
82	Range	-	-	1.21	0.12	0.11	CASTNET
83	Agric	-	-	1.43	0.13	0.14	CASTNET
84	Agric	-	-	1.17	0.09	0.08	CASTNET
85	Forest/Marsh	-	-	1.31	0.12	0.11	CASTNET
86	Forest	-	-	0.93	0.08	0.07	CASTNET
87	Desert	-	-	1.20	0.17	0.15	CASTNET
88	Forest	-	-	1.58	0.15	0.14	CASTNET
89	Forest	-	-	0.65	0.06	0.05	CASTNET
90	Agric	-	-	1.37	0.13	0.12	CASTNET
91	Range	-	-	1.36	0.20	0.21	CASTNET
92	Agric	-	-	1.56	0.13	0.12	CASTNET
93	Agric	-	-	1.30	0.13	0.12	CASTNET
94	Forest	-	-	1.33	0.13	0.12	CASTNET
95	Forest	-	-	2.49	0.24	0.25	CASTNET
96	Forest/Arid	-	-	1.53	0.16	0.16	CASTNET
97	Forest	-	-	0.99	0.10	0.10	CASTNET
98	Forest	-	-	1.24	0.11	0.09	CASTNET
99	Forest	-	-	1.20	0.11	0.11	CASTNET
100	Agric	-	-	1.45	0.11	0.11	CASTNET

101	Forest	-	-	1.05	0.08	0.06	CASTNET
102	Desert	-	-	1.09	0.19	0.18	CASTNET
103	Agric	-	-	1.26	0.11	0.10	CASTNET
104	Forest	-	-	0.86	0.07	0.07	CASTNET
105	Forest/Marsh	-	-	1.29	0.13	0.13	CASTNET
106	Agric	-	-	1.43	0.13	0.13	CASTNET
107	Forest	-	-	0.79	0.06	0.04	CASTNET
108	Forest	-	-	1.84	0.19	0.19	CASTNET
109	Forest	-	-	1.47	0.19	0.20	CASTNET
110	Forest	-	-	1.62	0.12	0.12	CASTNET
111	Range	-	-	1.48	0.16	0.17	CASTNET
112	Forest	-	-	1.31	0.11	0.10	CASTNET
113	Forest	-	-	1.14	0.11	0.08	CASTNET
114	Forest	-	-	0.67	0.05	0.04	CASTNET
115	Coastal/Marsh	-	-	1.09	0.08	0.08	CASTNET
116	Desert	-	-	1.47	0.19	0.18	CASTNET
117	Forest	-	-	0.91	0.09	0.08	CASTNET
118	Prairie	-	-	1.77	0.14	0.13	CASTNET
119	Forest	-	-	1.36	0.15	0.15	CASTNET
120	Forest	-	-	0.89	0.07	0.06	CASTNET
121	Forest	-	-	1.27	0.13	0.11	CASTNET
122	Agric	-	-	1.44	0.12	0.10	CASTNET
123	Agric	-	-	1.03	0.10	0.09	CASTNET
124	Agric	-	-	1.45	0.12	0.11	CASTNET
125	Agric	-	-	1.43	0.11	0.11	CASTNET
126	Forest	-	-	1.47	0.17	0.17	CASTNET

127	Forest	-	-	1.27	0.10	0.08	CASTNET
128	Forest	-	-	0.81	0.06	0.06	CASTNET
129	Forest	-	-	0.84	0.09	0.08	CASTNET
130	Forest	-	-	0.89	0.05	0.05	CASTNET
131	Agric	-	-	1.34	0.12	0.10	CASTNET
132	Prairie	-	-	2.17	0.19	0.18	CASTNET
133	Forest	-	-	1.30	0.12	0.10	CASTNET
134	Forest	-	-	1.10	0.11	0.10	CASTNET
135	Desert	-	-	1.65	0.17	0.17	CASTNET
136	Forest	-	-	1.27	0.14	0.14	CASTNET
137	Forest	-	-	1.48	0.14	0.14	CASTNET
138	Forest	-	-	1.53	0.14	0.15	CASTNET
139	Agric	-	-	1.25	0.11	0.09	CASTNET
140	Agric	-	-	1.33	0.11	0.10	CASTNET
141	Agric	-	-	1.63	0.12	0.12	CASTNET
142	Forest	-	-	1.62	0.19	0.19	CASTNET
143	Forest	-	-	1.71	0.20	0.20	CASTNET
144	Agric	-	-	1.37	0.11	0.10	CASTNET
145	Range	-	-	1.87	0.13	0.12	CASTNET
146	Forest	-	-	1.35	0.13	0.13	CASTNET
147	Forest	-	-	1.54	0.11	0.10	CASTNET
148	Forest	-	-	1.30	0.13	0.13	CASTNET
149	Agric	-	-	1.35	0.12	0.11	CASTNET
150	Agric	-	-	1.18	0.11	0.09	CASTNET
151	Agric	-	-	1.58	0.11	0.10	CASTNET
152	Forest	-	-	1.08	0.10	0.10	CASTNET

153	Forest	-	-	1.38	0.11	0.09	CASTNET
154	Agric	-	-	1.26	0.09	0.07	CASTNET
155	Agric	-	-	1.37	0.11	0.10	CASTNET
156	Forest	-	-	1.40	0.10	0.08	CASTNET
157	Agric	-	-	1.32	0.12	0.11	CASTNET
158	Forest	-	-	0.86	0.08	0.08	CASTNET
159	Prairie	-	-	1.94	0.18	0.16	CASTNET
160	Range	-	-	1.32	0.12	0.11	CASTNET
161	Forest	-	-	0.48	0.05	0.04	CASTNET
162	Agric	-	-	1.20	0.13	0.12	CASTNET
163	Forest	-	-	1.13	0.13	0.12	CASTNET

Additional references

Adon, M. *et al.* Dry deposition of nitrogen compounds (NO_2, HNO_3, NH_3), sulfur dioxide and ozone in west and central African ecosystems using the inferential method. *Atmospheric Chemistry and Physics* **13**, 11351-11374 (2013).

Adon, M. *et al.* Long term measurements of sulfur dioxide, nitrogen dioxide, ammonia, nitric acid and ozone in Africa using passive samplers. *Atmospheric Chemistry and Physics* **10**, 7467-7487 (2010).

Alejo, D. *et al.* Seasonal trends of atmospheric nitrogen dioxide and sulfur dioxide over North Santa Clara, Cuba. *Environmental monitoring and assessment* **185**, 6023-6033 (2013).

Andreae, M. O. *et al.* Optical properties and chemical composition of the atmospheric aerosol in urban Guangzhou, China. *Atmospheric Environment* **42**, 6335-6350 (2008).

Aneja, V. *et al.* Modeling Studies of Ammonia Dispersion and Dry Deposition at Some Hog Farms in North Carolina. *Journal of the Air & Waste Management Association* **58**, 1198-1207 (2008).

Bai, J. H., Wang, G. C., Meng, Z. Y. & Xu, X. B. Primary study on the characteristics of trace gas in clean area of North China. *Research of Environmental Sciences* **19**, 15-19 (2006).

Behera, S. N., Sharma, M., Aneja, V. P. & Balasubramanian, R. Ammonia in the atmosphere: a review on emission sources, atmospheric chemistry and deposition on terrestrial bodies. *Environmental science and pollution research international* **20**, 8092-8131 (2013).

Bytnerowicz, A., Fraczek, W., Schilling, S. & Alexander, D. Spatial and temporal distribution of ambient nitric acid and ammonia in the Athabasca Oil Sands Region, Alberta. *Journal of Limnology* **69**, 11-21 (2010).

Campos, V. P. *et al.* Chemical speciation and phase fractionation of N, S and Cl compounds in the atmosphere of Reconcavo, Bahia, Brazil. *Microchemical Journal* **109**, 58-67 (2013).

Campos, V. P., Cruz, L. P. S., Godoi, R. H. M., Godoi, A. F. L. & Tavares, T. M. Development and validation of passive samplers for atmospheric monitoring of SO_2, NO_2, O_3 and H_2S in tropical areas. *Microchemical Journal* **96**, 132-138 (2010).

Cao, J. J. *et al.* Characterization of Atmospheric Ammonia over Xi'an, China. *Aerosol and Air Quality Research* **9**, 277-289 (2009).

Carmichael, G. R. *et al.* Measurements of sulfur dioxide, ozone and ammonia concentrations in Asia, Africa, and South America using passive samplers. *Atmospheric Environment* **37**, 1293-1308 (2003).

Collett, K. S., Piketh, S. J. & Ross, K. E. An assessment of the atmospheric nitrogen budget on the South African Highveld. *South African Journal of Science* **106**, 1-9 (2010).

Cui, J., Zhou, J. & Yang, H. Atmospheric inorganic nitrogen in dry deposition to a typical red soil agro-ecosystem in southeastern China. *Journal of environmental monitoring : JEM* **12**, 1287-1294 (2010).

Cui, J., Zhou, J., Yang, H., Liang, J. N. & Liu, X. L. Quantitative input of atmospheric nitrogen to an agro-ecosystem in a typical red soil region. *ENVIRONMENTAL SCIENCE* **30**, 2221-2226 (2009).

Cui, J. *et al.* Atmospheric NO_2 and NH_3 deposition into a typical agro-ecosystem in Southeast China. *Journal of environmental monitoring : JEM* **13**, 3216-3221 (2011).

Delon, C. *et al.* Nitrogen compounds emission and deposition in West African ecosystems: comparison between wet and dry savanna. *Biogeosciences* **9**, 385-402 (2012).

Delon, C. *et al.* Atmospheric nitrogen budget in Sahelian dry savannas. *Atmospheric Chemistry and Physics* **10**, 2691-2708 (2010).

Deng, J. J., Wang, T. J., Li, S., Xie, M. & Fan, J. L. Study on atmospheric nitrogen oxidant and deposition flux in suburban of Nanjing. *Scientia Meteorologica Sinica* **29**, 25-30 (2009).

Duan, F. K. *et al.* Concentration and chemical characteristics of $PM_{2.5}$ in Beijing, China: 2001-2002. *Sci Total Environ* **355**, 264-275 (2006).

Endo, T. *et al.* Regional characteristics of dry deposition of sulfur and nitrogen compounds at EANET sites in Japan from 2003 to 2008. *Atmospheric Environment* **45**, 1259-1267 (2011).

Fan, J. L., Hu, Z. Y. Dynamics of atmospheric NO_2 concentration in a forest eco-system at Yingtan, Jiangxi Province. *China Enviromental Science* **26**, 171-175 (2006).

Fan, J. L., Hu, Z. Y., Wang, T. J. & Zhou, J. Dynamics of dry deposition velocities of atmospheric nitrogen compounds in a broadleaf forestland. *China Environmental Science* **29**, 574-577 (2009).

Fan, J. L. *et al.* Atmospheric inorganic nitrogen deposition to a typical red soil forestland in southeastern China. *Environmental monitoring and assessment* **159**, 241-253 (2009).

Fan, J. L., Hu, Z. Y., Zhou, J. & Wu, C. Y. H. Comparative study on the observation of atmospheric nitrogen deposition in a forestland. *China Environmental Science* **33**, 786-792 (2013).

Fan, J. L. *et al.* Observation of atmospheric nitrogen deposition into forestland. *China Environmental Science* **27**, 7-9 (2007).

Fattore, F. *et al.* Seasonal trends of dry and bulk concentration of nitrogen compounds over a rain forest in Ghana. *Biogeosciences* **11**, 3069-3081 (2014).

Flechard, C. R. *et al.* Dry deposition of reactive nitrogen to European ecosystems: a comparison of inferential models across the NitroEurope network. *Atmospheric Chemistry and Physics* **11**, 2703-2728 (2011).

Gibson, M. D. *et al.* The spatial and seasonal variation of nitrogen dioxide and sulfur dioxide in Cape Breton Highlands National Park, Canada, and the association with lichen abundance. *Atmospheric Environment* **64**, 303-311 (2013).

Guo, Y.-t., Zhang, J., Wang, S.-g., She, F. & Li, X. Long-term characterization of major water-soluble inorganic ions in PM_{10} in coastal site on the Japan Sea. *Journal of Atmospheric Chemistry* **68**, 299-316 (2012).

He, J. Comparison of NO_2 and SO_2 Measurements Using Different Passive Samplers in Tropical Environment. *Aerosol and Air Quality Research*, **14**, 355-363 (2014).

He, J. *et al.* Dry and wet atmospheric deposition of nitrogen and phosphorus in Singapore. *Atmospheric Environment* **45**, 2760-2768 (2011).

He, K. B. *et al.* The characteristics of $PM_{2.5}$ in Beijing, China. *Atmospheric Environment* **35**, 4959-4970 (2001).

Hien, P. D., Hangartner, M., Fabian, S. & Tan, P. M. Concentrations of NO_2, SO_2, and benzene across Hanoi measured by passive diffusion samplers. *Atmospheric Environment* **88**, 66-73 (2014).

Ho, K. F. *et al.* Characterization of chemical species in $PM_{2.5}$ and PM_{10} aerosols in Hong Kong. *Atmospheric Environment* **37**, 31-39 (2003).

Hole, L. R., Brunner, S. H., Hanssen, J. E. & Zhang, L. Low cost measurements of nitrogen and sulphur dry deposition velocities at a semi-alpine site: gradient measurements and a comparison with deposition model estimates. *Environmental pollution* **154**, 473-481 (2008).

Hu, G. *et al.* Variability, formation and acidity of water-soluble ions in $PM_{2.5}$ in Beijing based on the semi-continuous observations. *Atmospheric Research* **145-146**, 1-11 (2014).

Hu, Q., Zhang, L., Evans, G. J. & Yao, X. Variability of atmospheric ammonia related to potential emission sources in downtown Toronto, Canada. *Atmospheric Environment* **99**, 365-373 (2014).

Huang, B. *et al.* Chemical composition, diurnal variation and sources of $PM_{2.5}$ at two industrial sites of South China. *Atmospheric Pollution Research*, **4**, 298-305 (2013).

Huang, Y. K. *et al.* Land use patterns and SO_2 and NO_2 pollution in Ulaanbaatar, Mongolia. *Environmental research* **124**, 1-6 (2013).

Hueglin, C. *et al.* Chemical characterisation of $PM_{2.5}$, PM_{10} and coarse particles at urban, near-city and rural sites in Switzerland. *Atmospheric Environment* **39**, 637-651 (2005).

Ianniello, A. *et al.* Chemical characteristics of inorganic ammonium salts in $PM_{2.5}$ in the atmosphere of Beijing (China). *Atmospheric Chemistry and Physics* **11**, 10803-10822 (2011).

Ianniello, A. *et al.* Occurrence of gas phase ammonia in the area of Beijing (China). *Atmospheric Chemistry and Physics* **10**, 9487-9503 (2010).

Laakso, L. *et al.* Basic characteristics of atmospheric particles, trace gases and meteorology in a relatively clean Southern African Savannah environment. *Atmospheric Chemistry and Physics* **8**, 4823-4839 (2008).

Laakso, L. *et al.* South African EUCAARI measurements: seasonal variation of trace gases and aerosol optical properties. *Atmospheric Chemistry and Physics* **12**, 1847-1864 (2012).

Latif, M. T. *et al.* Long term assessment of air quality from a background station on the Malaysian Peninsula. *Sci Total Environ* **482-483**, 336-348 (2014).

Li, K. H. *et al.* Atmospheric nitrogen deposition at two sites in an arid environment of central Asia. *Plos One* **8**, e67018. doi:10.1371/journal.pone.0067018.g001 (2013).

Li, K. H. *et al.* Atmospheric reactive nitrogen concentrations at ten sites with contrasting land use in an arid region of central Asia. *Biogeosciences* **9**, 4013-4021 (2012).

Li, X. *et al.* Chemical composition and size distribution of airborne particulate matters in Beijing during the 2008 Olympics. *Atmospheric Environment* **50**, 278-286 (2012).

Li, Y. *et al.* Observations of ammonia, nitric acid, and fine particles in a rural gas production region. *Atmospheric Environment* **83**, 80-89 (2014).

Lin, W. Characteristics of gaseous pollutants at Jinsha, a remote mountain site in Central China. *SCIENTIA SINICA Chimica* **41**, 136-144 (2011).

Lin, W., Xu, X., Ge, B. & Zhang, X. Characteristics of gaseous pollutants at Gucheng, a rural site southwest of Beijing. *Journal of Geophysical Research* **114** (2009).

Lin, W., Xu, X., Sun, J., Liu, X. & Wang, Y. Background concentrations of reactive gases and the impacts of long-range transport at the Jinsha regional atmospheric background station. *Science China Earth Sciences* **54**, 1604-1613 (2011).

Lin, W., Xu, X., Yu, X., Zhang, X. & Huang, J. Observed levels and trends of gaseous SO_2 and HNO_3 at Mt. Waliguan, China: Results from 1997 to 2009. *Journal of Environmental Sciences* **25**, 726-734 (2013).

Lourens, A. S. *et al.* Spatial and temporal assessment of gaseous pollutants in the Highveld of South Africa. *South African Journal of Science* **107**, 1-8 (2011).

Luo, X. S. *et al.* An evaluation of atmospheric Nr pollution and deposition in North China after the Beijing Olympics. *Atmospheric Environment* **74**, 209-216 (2013).

Mallik, C. & Lal, S. Seasonal characteristics of SO_2, NO_2, and CO emissions in and around the Indo-Gangetic Plain. *Environmental monitoring and assessment* **186**, 1295-1310 (2014).

Marner, B. B. & Harrison, R. M. A spatially refined monitoring based study of atmospheric nitrogen deposition. *Atmospheric Environment* **38**, 5045-5056 (2004).

Martins, J. J., Dhammapala, R. S., Lachmann, G., Galy-Lacaux, C. & Pienaar, J. J. Long-term

measurements of sulphur dioxide, nitrogen dioxide, ammonia, nitric acid and ozone in southern Africa using passive samplers. *South African Journal of Science* **103**, 336-342 (2007).

Matsuda, K. Estimation of dry deposition for sulfur and nitrogen compounds in the atmosphere-Updated parameterization of deposition velocity. *Journal of Japanese Society for Atmospheric Environment* **43**, 332–339 (2008). (in Japanese with English abstract)

Meng, Z. Seasonal Variation of Ammonia and Ammonium Aerosol at a Background Station in the Yangtze River Delta Region, China. *Aerosol and Air Quality Research* **14**, 756-766 (2014).

Meng, Z. Y., Jia, X. F., Zhang, R. J., Yu, X. M. & Ma, Q. L. Characteristics of $PM_{2.5}$ at Lin'an regional background station in the Yangtze River Delta Region. *Journal of Applied Meteorological Science* **23**, 424-432 (2012).

Meng, Z. Y. *et al.* Characteristics of atmospheric ammonia over Beijing, China. *Atmospheric Chemistry and Physics* **11**, 6139-6151 (2011).

Meng, Z. Y. *et al.* Characteristics of trace gaseous pollutants at a regional background station in Northern China. *Atmospheric Chemistry and Physics* **9**, 927-936 (2009).

Meng, Z.-Y. *et al.* Ambient sulfur dioxide, nitrogen dioxide, and ammonia at ten background and rural sites in China during 2007–2008. *Atmospheric Environment* **44**, 2625-2631 (2010).

Myles, L., Meyers, T. P. & Robinson, L. Relaxed eddy accumulation measurements of ammonia, nitric acid, sulfur dioxide and particulate sulfate dry deposition near Tampa, FL, USA. *Environmental Research Letters* **2**, 034004 (2007).

Neirynck, J. *et al.* Fluxes of oxidised and reduced nitrogen above a mixed coniferous forest exposed to various nitrogen emission sources. *Environmental pollution* **149**, 31-43 (2007).

Pan, Y. P., Wang, Y. S., Tang, G. Q. & Wu, D. Wet and dry deposition of atmospheric nitrogen at ten sites in Northern China. *Atmospheric Chemistry and Physics* **12**, 6515-6535 (2012).

Qi, J. H., Shi, J. H., Gao, H. W. & Sun, Z. Atmospheric dry and wet deposition of nitrogen species and its implication for primary productivity in coastal region of the Yellow Sea, China. *Atmospheric Environment* **81**, 600-608 (2013).

Salem, A. A., Soliman, A. A. & El-Haty, I. A. Determination of nitrogen dioxide, sulfur dioxide, ozone, and ammonia in ambient air using the passive sampling method associated with ion chromatographic and potentiometric analyses. *Air quality, atmosphere, & health* **2**, 133-145 (2009).

Schrader, F. & Brummer, C. Land Use Specific Ammonia Deposition Velocities: a Review of Recent Studies (2004-2013). *Water Air Soil Pollut* **225**, 2114, doi:10.1007/s11270-014-2114-7 (2014).

Sharma, S. K. *et al.* Characteristics of ambient ammonia over Delhi, India. *Meteorology and Atmospheric Physics* **124**, 67-82 (2013).

Sharma, S. K. *et al.* Measurement of ambient NH_3 over Bay of Bengal during W_ICARB Campaign. *Annales Geophysicae* **30**, 371-377 (2012).

Shen, J. *et al.* Atmospheric dry and wet nitrogen deposition on three contrasting land use types of an agricultural catchment in subtropical central China. *Atmospheric Environment* **67**, 415-424 (2013).

Shen, J. *et al.* Atmospheric ammonia and particulate ammonium from agricultural sources in the North China Plain. *Atmospheric Environment* **45**, 5033-5041 (2011).

Shen, J. L. *et al.* High concentrations and dry deposition of reactive nitrogen species at two sites in the North China Plain. *Environmental pollution* **157**, 3106-3113 (2009).

Singh, S. & Kulshrestha, U. C. Abundance and distribution of gaseous ammonia and particulate ammonium at Delhi, India. *Biogeosciences* **9**, 5023-5029 (2012).

Song, S. *et al.* Chemical characteristics of size-resolved $PM_{2.5}$ at a roadside environment in Beijing, China. *Environmental pollution* **161**, 215-221 (2012).

Spataro, F. *et al.* Occurrence of atmospheric nitrous acid in the urban area of Beijing (China). *Sci Total Environ* **447**, 210-224 (2013).

Staelens, J. *et al.* Trends in atmospheric nitrogen and sulphur deposition in northern Belgium. *Atmospheric Environment* **49**, 186-196 (2012).

Su, H., Zhu, B., Yan, X. Y. & Yang, R. Numerical simulation for dry deposition of ammonia and nitrogen dioxide in a small watershed in Jurong county of Jiangsu province. *Chinese Journal of Agrometeorology* **30**, 335-342 (2009).

Sun, Y. *et al.* Characterization of summer organic and inorganic aerosols in Beijing, China with an Aerosol Chemical Speciation Monitor. *Atmospheric Environment* **51**, 250-259 (2012).

Sun, Y. *et al.* The air-borne particulate pollution in Beijing—concentration, composition, distribution and sources. *Atmospheric Environment* **38**, 5991-6004 (2004).

Tao, Y., Yin, Z., Ye, X., Ma, Z. & Chen, J. Size distribution of water-soluble inorganic ions in urban aerosols in Shanghai. *Atmospheric Pollution Research* **5**, 639-647 (2014).

Ti, C. P. Regional nitrogen budget at different spatial scales. *PhD thesis, Nanjing Agricultural University* (2011).

Trebs, I. *et al.* Dry and wet deposition of inorganic nitrogen compounds to a tropical pasture site (Rondônia, Brazil). *Atmospheric Chemistry and Physics* **6**, 447-469 (2006).

Wang, G. H. *et al.* Impact of Gobi desert dust on aerosol chemistry of Xi'an, inland China during spring 2009: differences in composition and size distribution between the urban ground surface and the mountain atmosphere. *Atmospheric Chemistry and Physics* **13**, 819-835 (2013).

Wang, S. *et al.* Observation of NO_3 radicals over Shanghai, China. *Atmospheric Environment* **70**, 401-409 (2013).

Wang, T. J., Liu, Q., Zhao, H., Zhou, J. & Fan, J. L. Atmospheric nitrogen deposition in agrecosystem in red soil region of Jiangxi province. *Acta Pedologica Sinica* **45**, 280-287 (2008).

Wang, X. *et al.* Atmospheric nitrogen deposition to forest and estuary environments in the Pearl River Delta region, southern China. *Tellus B* **65**, 20480 (2013).

Wei, Y. Atmospheric nitrogen deposition and acid rain in different ecological regions in Shaanxi Province. *MS thesis, Northwest A & F University* (2011).

Wei, Z., Wang, L.-T., Chen, M.-Z. & Zheng, Y. The 2013 severe haze over the southern Hebei, China: $PM_{2.5}$ composition and source apportionment. *Atmospheric Pollution Research* **5**, 759-768 (2014).

Wen, D., Zhang, L., Lin, J. C., Vet, R. & Moran, M. D. An evaluation of ambient ammonia concentrations over southern Ontario simulated with different dry deposition schemes within STILT-Chem v0.8. *Geoscientific Model Development* **7**, 1037-1050 (2014).

Xiang, R. J. *et al.* Study of the chemical characteristics of dry deposition in typical acid rain areas of China. *Journal of Hunan University (Natural Sciences)* **38**, 67-71 (2011).

Xu, J. *et al.* Characteristics of water soluble ionic species in fine particles from a high altitude site on the northern boundary of Tibetan Plateau: Mixture of mineral dust and anthropogenic aerosol. *Atmospheric Research* **143**, 43-56 (2014).

Yang, R., Hayashi, K., Zhu, B., Li, F. & Yan, X. Atmospheric NH_3 and NO_2 concentration and nitrogen deposition in an agricultural catchment of Eastern China. *Sci Total Environ* **408**, 4624-4632 (2010).

Yao, X. H. *et al.* The water-soluble ionic composition of $PM_{2.5}$ in Shanghai and Beijing, China.

Atmospheric Environment **36**, 4223-4234 (2002).

Yao, X. H. & Zhang, L. Analysis of passive-sampler monitored atmospheric ammonia at 74 sites across southern Ontario, Canada. *Biogeosciences* **10**, 7913-7925 (2013).

Ye, B. M. *et al.* Concentration and chemical composition of $PM_{2.5}$ in Shanghai for a 1-year period. *Atmospheric Environment* **37**, 499-510 (2003).

Yu, D. J., Wu, Y. L., Song, Q. L., Dai, X. & Lin, W. L. Environmental Characteristics and Its Observations at Longfengshan WMO Regional Atmospheric Background Station. *Climate Change Research Letters* **01**, 65-73 (2012).

Zbieranowski, A. L. & Aherne, J. Ambient concentrations of atmospheric ammonia, nitrogen dioxide and nitric acid across a rural–urban–agricultural transect in southern Ontario, Canada. *Atmospheric Environment* **62**, 481-491 (2012).

Zbieranowski, A. L. & Aherne, J. Spatial and temporal concentration of ambient atmospheric ammonia in southern Ontario, Canada. *Atmospheric Environment* **62**, 441-450 (2012).

Zhang, H. *et al.* Source apportionment of PM2.5 nitrate and sulfate in China using a source-oriented chemical transport model. *Atmospheric Environment* **62**, 228-242 (2012).

Zhang, L. *et al.* Nitrogen deposition to the United States: distribution, sources, and processes. *Atmospheric Chemistry and Physics* **12**, 4539-4554 (2012).

Zhang, L. *et al.* Dry deposition of individual nitrogen species at eight Canadian rural sites. *Journal of Geophysical Research* **114**, D02301 (2009).

Zhang, R. *et al.* Chemical characterization and source apportionment of $PM_{2.5}$ in Beijing: seasonal perspective. *Atmospheric Chemistry and Physics* **13**, 7053-7074 (2013).

Zhang, W. *et al.* Analysis on input of atmospheric nitrogen dry deposition in Urumqi. *Arid Zone Research* **28**, 710-716 (2011).

Zhang, Y., Wang, T. J., Hu, Z. Y. & Xu, C. K. Temporal variety and spatial distribution of dry deposition velocities of typical air pollutants over different landuse types. *Climatic and Environmental Research* **9**, 591-604 (2004).

Zhang, Y.-F. *et al.* The study on vertical variability of PM_{10} and the possible sources on a 220 m tower, in Tianjin, China. *Atmospheric Environment* **45**, 6133-6140 (2011).

Zhao, P. S. *et al.* Characteristics of concentrations and chemical compositions for $PM_{2.5}$ in the region of Beijing, Tianjin, and Hebei, China. *Atmospheric Chemistry and Physics* **13**, 4631-4644 (2013).

Zhou, J., Cui, J., Fan, J. L., Liang, J. N. & Wang, T. J. Dry deposition velocity of atmospheric nitrogen in a typical red soil agro-ecosystem in Southeastern China. *Environmental monitoring and assessment* **167**, 105-113 (2010).

Zhu, L., Chen, Y., Guo, L. & Wang, F. Estimate of dry deposition fluxes of nutrients over the East China Sea: The implication of aerosol ammonium to non-sea-salt sulfate ratio to nutrient deposition of coastal oceans. *Atmospheric Environment* **69**, 131-138 (2013).

Zimmermann, F., Plessow, K., Queck, R., Bernhofer, C. & Matschullat, J. Atmospheric N- and S-fluxes to a spruce forest—Comparison of inferential modelling and the throughfall method. *Atmospheric Environment* **40**, 4782-4796 (2006).
